# Quantitative assessment of inter-observer consistency in target volume delineation following breast-conserving surgery and mastectomy

**DOI:** 10.3389/fonc.2026.1850269

**Published:** 2026-07-16

**Authors:** An Yi, Lihong He, Zijie Wang, Xin Liu, Bin Li, Zhuoyu He, Jin Liu, Chunling Jiang

**Affiliations:** 1 Jiangxi Medical College, Nanchang University, Nanchang, China; 2 Department of Radiation Oncology, Jiangxi Cancer Hospital & Institute, Jiangxi Clinical Research Center for Cancer, The Second Affiliated Hospital of Nanchang Medical College, Nanchang, China; 3 Department of Radiation Oncology, Shanghai General Hospital, Shanghai Jiao Tong University School of Medicine, Shanghai, China; 4 Yichun Vocational Technical College of Medicine, The Affiliated Hospital of Yichun Vocational Technical College, Yichun, China

**Keywords:** breast cancer, clinical target volume, dice similarity coefficient, hausdorff distance, radiotherapy

## Abstract

**Background:**

Consistent clinical target volume (CTV) delineation is vital for breast cancer radiotherapy. While organs-at-risk definitions are increasingly automated, manual CTV delineation of complex post-surgical volumes remains highly observer-dependent, potentially compromising treatment homogeneity.

**Objective:**

To quantify inter-observer discrepancies in postoperative breast cancer CTV contouring among regional oncologists and to qualitatively analyze the reasons for these contouring discrepancies to promote standardization and quality assurance.

**Methods:**

Based on CT datasets of two representative post-surgical breast cancer cases (BCS and MRM), 22 frontline radiation oncologists from regional municipal hospitals independently contoured the required CTVs. Inter-observer contouring discrepancies across these manual target volumes were quantitatively evaluated via Dice similarity coefficients (DSC) and Hausdorff distances (HD) against a consensus reference standard established through a two-expert consensus workflow.

**Results:**

A total of 20 completed datasets, each for both the BCS and MRM cases, were successfully retrieved and analyzed. The large volumes achieved the highest overlap (*CTVp_breast* and *CTVp_thoracic wall* DSC mean: 0.80 ± 0.07 and 0.63 ± 0.08, respectively), though mean 95% Hausdorff distances (HD_95_) were elevated at 42.84 ± 50.62 mm and 45.73 ± 60.44 mm. Post-BCS focal volumes (*CTV_tb* and *CTV_boost*) showed intermediate overlap (both DSC mean: 0.56) but restricted boundary deviations (mean HD_95_: 11.51 mm and 19.94 mm). For nodal stations, consistency was moderate for the supraclavicular volume (*CTVn_L4* DSC mean: 0.57 ± 0.15, HD_95_: 26.21 ± 15.08 mm) but dropped substantially for axillary Level III (*CTVn_L3* DSC mean: 0.38 ± 0.26, HD_95_: 37.95 ± 37.94 mm) and internal mammary nodes (*CTVn_IMN* DSC mean: 0.42 ± 0.14, HD_95_: 42.67 ± 25.70 mm).

**Conclusion:**

Delineation consistency is acceptable for large volumes (*CTVp_breast* and *CTVp_thoracic wall*) but poor for regional nodal stations, demonstrating that standardized anatomical training is crucial to improve radiotherapy uniformity.

## 1 Introduction

Breast cancer is the most common malignancy and a leading cause of cancer-related mortality among women worldwide (1, 2). According to the latest national cancer registry statistics released by the National Cancer Center, it ranks as the second most common female malignancy in China, with annual new cases reaching approximately 357,200 and causing over 75,000 deaths (3). Given this substantial and growing disease burden, effective locoregional treatment is essential. Postoperative radiotherapy plays a critical role in improving local tumor control, reducing the risk of locoregional recurrence and distant metastasis, and prolonging overall survival (OS) (4, 5).

Target volume delineation is a fundamental component of radiation therapy planning, as the geometric consistency of clinical target volumes (CTVs) and organs at risk (OARs) directly influences dosimetric precision and therapeutic efficacy (6, 7). To address the time-intensive nature and inherent subjectivity of manual contouring, deep learning (DL)-based auto-segmentation algorithms have been developed and have demonstrated the capability to generate reliable CTV and OAR contours for breast cancer, thereby enhancing clinical workflow efficiency (8–10). Nevertheless, manual contouring by experienced radiation oncologists remains the clinical consensus standard, and AI-generated contours still require expert review and modification before clinical use (6, 10). Consequently, understanding and reducing inter-observer variability in manual CTV contouring remains a clinical priority.

While inter-observer variability in breast CTV contouring is documented in high-volume academic centers, data reflecting radiation oncologists in non-specialized, community-based settings—who treat the vast majority of patients in developing countries—are less common (11, 12). These regional institutions often lack standardized subspecialty training and routine quality assurance. To address this gap, 22 radiation oncologists from municipal hospitals across Jiangxi Province independently contoured target volumes. Rather than replicating broad consensus data, this study analyzes the common error types and the reasons for their occurrence following both breast-conserving surgery (BCS) and modified radical mastectomy (MRM) scenarios, providing an empirical basis for standardizing regional clinical practice.

## 2 Materials and methods

### 2.1 Patient selection

Two representative right-sided breast cancer patients treated at Jiangxi Cancer Hospital were selected: a stage I (*pT1N0M0, Luminal A*) case post-breast-conserving surgery (BCS), and a stage IIB (*pT2N1M0, Luminal B*) case post-modified radical mastectomy (MRM) with the primary tumor medially located. Both patients were simulated in a supine position using customized cervicothoracic boards and thermoplastic masks. Radio-opaque lead wires demarcated outer breast contours and surgical scars. CT scanning extended from the superior margin of the C1 vertebra to the inferior margin of the L2 vertebra with a 5 mm slice thickness.

### 2.2 Target definition and delineation

For the post-BCS case (*pT1N0M0*), the defined target volumes encompassed the tumor bed (*CTV_tb*), the tumor bed boost volume (*CTV_boost*), and the residual whole breast (*CTVp_breast*) based on standard indications. For the post-MRM case (*pT2N1M0*) with a complete axillary lymph node dissection (ALND) and a medially located tumor, the target volumes requiring mandatory delineation included the affected chest wall (*CTVp_thoracic wall*), supraclavicular lymph node station (*CTVn_L4*), axillary level III (*CTVn_L3*), and internal mammary node chain (*CTVn_IMN*). Because elective irradiation of axillary levels I and II (*CTVn_L1* and *CTVn_L2*) is not routinely recommended following a comprehensive ALND in this specific scenario, they were explicitly excluded from the required target portfolio. Organs at risk (OARs) contoured across all datasets included the heart (incorporating the left anterior descending artery [LAD]), bilateral lungs, contralateral breast, spinal cord, liver, stomach, thyroid, and esophagus.

These Target volume and OAR delineations generally followed the ESTRO consensus guidelines (13). However, the ventral boundary of the post-MRM chest wall (CTVp_thoracic) was defined strictly at the skin surface (Skin) according to the RTOG atlas (14), rather than the ESTRO-recommended 5 mm sub-skin margin (13). This adaptation accommodates the typically thinner subcutaneous adipose tissue in East Asian women, minimizing the clinical risk of under-dosing high-risk dermal and subcutaneous lymphatic networks (15). The specific anatomical boundaries applied for each post-surgical clinical target volume (CTV) are systematically structured as the formalized delineation standards in **Table 1**. Deviations arising solely from the systematic RTOG-ESTRO guideline difference at the anterior margin were not scored as contouring errors.

**Table 1:** Delineation standards for post-BCS and post-MRM clinical target volumes (CTVs).

Clinical target volume (CTV)	Cranial	Caudal	Ventral	Dorsal	Medial	Lateral
Post-BCS volume						
Residual breast CTVp_breast	Upper border of palpable/visible breast tissue; maximally up to the inferior edge of the sterno-clavicular joint)	Most caudal CT slice with visible breast shape.	5 mm beneath the skin surface.	The major pectoral muscle, or the costae and intercostal muscles where no muscle	Lateral to the medial perforating mammary vessels; maximally to the edge of the sternal bone	Lateral breast fold, anterior to the lateral thoracic artery
Tumor Bed CTV_tb & CTV_boost	—	—	—	—	—	—
Post-MRM volumes						
Thoracic wall CTVp_thoracic wall	Guided by palpable/visible signs, maximally up to the inferior edge of the sterno-clavicular joint.	Guided by palpable/visible signs; if appropriate, guided by the contralateral breast.	Skin(RTOG)	The major pectoral muscle, or the costae and intercostal muscles where no muscle	Guided by palpable/visible signs; if appropriate, guided by the contralateral breast.	Guided by palpable/visible signs, usually anterior to the mid-axillary line
Internal mammary chain CTVn_IMN	Caudal limit of CTVn_L4	Cranial side of the 4th rib (in selected cases 5th rib in certain cases)	Ventral limit of the vascular area	Pleura	5 mm from the internal mammary vein and artery	5 mm from the internal mammary vein and artery
Axilla level 3 CTVn_L3	Cranial extent of the subclavian artery (i.e., 5 mm cranial of the subclavian vein)	5 mm caudal to the subclavian vein. If appropriate: top of surgical ALND	Major pectoral muscle	Up to 5 mm dorsal of the subclavian vein or to the costae and intercostal muscles	Junction of subclavian and internal jugular veins –> level 4	Medial side of the minor pectoral muscle
Axilla level 4 CTVn_L4	Includes the cranial extent of the subclavian artery (i.e., 5 mm cranial to the subclavian vein)	Includes the subclavian vein with a 5 mm margin, thus connecting to the cranial border of CTVn_IMN	Sternocleidomastoid muscle, dorsal edge of the clavicle	Pleura	Including the jugular vein without margin; excluding the thyroid gland and the common carotid artery	Includes the anterior scalene muscles and connects to the medial border of CTVn_L3

Focal Volumes (*CTV_tb* & *CTV_boost*): Comprehensively defined based on preoperative imaging, surgical clips, seroma, and scar markers; *CTV_boost* is generated via a uniform 1 cm isotropic expansion from the TB. Due to their patient-specific spherical/focal nature, they do not follow static independent planar 6-directional anatomical borders.

To ensure that geometric variations were derived strictly from independent clinical judgment within these conceptual frameworks, all OARs were auto-segmented via the RT-Mind platform (Beijing Yizhiying Technology Co., Ltd., Beijing, China) and locked as a uniform, unmodifiable background template. This platform implements an advanced, customized Convolutional Neural Network architecture termed RTD-Net, which is optimized from the classic U-Net framework to execute deep-learning-based automated organ segmentations. Consequently, participating physicians focused exclusively on manual CTV delineation from scratch on the integrated RT-Mind contouring workstation. Crucially, before contouring, participants were directed to follow the ESTRO guidelines but were deliberately not provided with a predefined target list; they had to independently judge which specific CTV substructures were clinically indicated based on the presented case profiles. To capture a realistic snapshot of regional community oncology practice, the participating cohort comprised 22 junior- to mid-level frontline radiation oncologists from municipal hospitals. A senior radiation oncologist performed the initial CTV delineation, which was subsequently reviewed and validated by a second senior expert to establish the consensus reference standard for all comparative analyses.

### 2.3 Evaluation metrics

The contouring agreement of each participating physician was quantitatively evaluated against the expert-validated consensus reference standard using two complementary spatial metrics (16, 17):

#### 2.3.1 Dice similarity coefficient

The DSC measures the degree of volumetric overlap between two segmented regions. The formula is:


$$DSC=2\frac{|A\;\cap \;B\;|}{|A|+|B|}$$

Where *A* represents the consensus reference standard volume, and *B* represents the volume delineated by the evaluated physician. DSC values range from 0 (no overlap) to 1 (perfect agreement), with higher values indicating superior delineation consistency.

#### 2.3.2 Hausdorff distance

The HD quantifies the maximum surface mismatch between two contour point sets in three-dimensional space. The formula is:


HD(A,B)=max[h(A,B),h(B,A)]

$$h(A,B)={\text{max}}_{\text{x}\in \text{A}}{\text{ min}}_{\text{y}\in \text{B}}∥x-y∥$$

$$h(\text{B},\text{A})={\text{max}}_{\text{y}\in \text{B}}{\text{ min}}_{\text{x}\in \text{A}}∥\text{y}-\text{x}∥$$

Where *h*(*A*, *B*) denotes the maximum of the shortest distances from each point in set A to set B, and h(B, A) conversely represents the directed distance from point set B to point set A. Because the standard HD is highly sensitive to single outlier points, we report the 95th and 98th percentiles of the HD distribution (HD_95_ and HD_98_), which provide more robust measures of boundary conformity. Smaller HD values indicate tighter spatial agreement between contours.

### 2.4 Statistical analysis

Statistical analyses were performed using SPSS version 26.0 (IBM Corp., Armonk, NY, USA). Given the descriptive nature of this study and the limited sample size, formal inferential statistical comparisons were not performed. To accurately characterize the central tendency and dispersion of the delineation metrics under potential non-normal distributions, continuous variables were expressed as mean ± standard deviation (SD), accompanied by the median and interquartile range (IQR).

## 3 Results

### 3.1 Completion rates

For the post-BCS case, 22 datasets were initially collected. Two were excluded because the participants contoured *CTVp_breast* (with inherent outlier DSC values of 0.04 and 0.05) while omitting all other mandatory structures, leaving 20 datasets for final analysis. The completion rate was 100% (20/20) for *CTVp_breast*, and 85.0% (17/20) each for *CTV_tb* and *CTV_boost*.

For the post-MRM case, 22 participants initially entered the study; one withdrew mid-way, and another was excluded because the participant contoured exclusively *CTVp_thoracic wall* (with an inherent outlier DSC of 0.15) and omitted all remaining structures, leaving 20 datasets for final evaluation. The completion rate was 100% (20/20) for *CTVp_thoracic wall*, but dropped to 70.0% (14/20) for *CTVn_L4*, 60.0% (12/20) for *CTVn_L3*, and only 30.0% (6/20) for *CTVn_IMN*.

### 3.2 Quantitative results

The quantitative evaluation metrics for postoperative breast cancer target volume delineation are summarized in **Tables 2**, **3**.

**Table 2:** Quantitative assessment of inter-observer consistency in target volume delineation following breast-conserving surgery (BCS).

Evaluation metrics	DSC		HD_95_		HD_98_	
	Mean ± SD	Median (IQR)	Mean ± SD	Median (IQR)	Mean ± SD	Median (IQR)
CTVp_breast (N = 20)	0.80 ± 0.07	0.82 (0.76-0.86)	42.84 ± 50.62	22.50 (11.97-36.47)	61.48 ± 79.77	25.46 (20.00-55.02)
CTV_tb (N = 17)	0.56 ± 0.19	0.63 (0.45-0.67)	11.51 ± 6.69	8.65 (6.63-15.87)	14.03 ± 9.42	10.83 (7.88-17.74)
CTV_boost (N = 17)	0.56 ± 0.17	0.59 (0.43-0.71)	19.94 ± 18.23	13.07 (9.56-25.45)	21.76 ± 18.91	14.08 (10.90-28.07)

Values are presented as either Mean ± Standard Deviation (SD) or Median (Interquartile Range, IQR) as indicated. DSC, Dice similarity coefficient; HD_95_, 95th-percentile Hausdorff distance; HD_98_, 98th-percentile Hausdorff distance. HD values are reported in millimeters (mm). N represents the structure-specific sample size.

**Table 3:** Quantitative assessment of inter-observer consistency in target volume delineation following modified radical mastectomy (MRM).

Evaluation metrics	DSC		HD_95_		HD_98_	
	Mean ± SD	Median (IQR)	Mean ± SD	Median (IQR)	Mean ± SD	Median (IQR)
CTVp_thoracic wall (N = 20)	0.63 ± 0.08	0.63 (0.57-0.71)	45.73 ± 60.44	22.54 (10.68-65.75)	53.97 ± 66.57	27.50 (14.03-73.75)
CTVn_IMN (N = 6)	0.42 ± 0.14	0.46 (0.34-0.51)	42.67 ± 25.70	35.00 (20.41-66.37)	54.19 ± 28.22	47.50 (28.75-83.79)
CTVn_L4 (N = 14)	0.57 ± 0.15	0.61 (0.45-0.72)	26.21 ± 15.08	23.17 (13.75-37.26)	30.08 ± 17.06	28.58 (14.34-40.92)
CTVn_L3 (N = 12)	0.38 ± 0.26	0.43 (0.09-0.62)	37.95 ± 37.94	23.50 (15.99-48.84)	42.06 ± 37.76	31.27 (18.72-53.67)

Values are presented as either Mean ± Standard Deviation (SD) or Median (Interquartile Range, IQR) as indicated. DSC, Dice similarity coefficient; HD_95_, 95th-percentile Hausdorff distance; HD_98_, 98th-percentile Hausdorff distance. HD values are reported in millimeters (mm). N represents the structure-specific sample size.

For the post-BCS case (**Table 2**), the whole breast CTV (CTVp_breast) demonstrated the highest inter-observer agreement, with a mean DSC of 0.80 ± 0.07. In contrast, both the CTV_boost mean DSC = 0.56 ± 0.17 and the CTV_tb mean DSC = 0.56 ± 0.19 showed substantially lower consistency. However, the HD metrics for these tumor bed targets were notably smaller than those of the whole breast. Conversely, the whole breast CTV exhibited large HD values accompanied by substantial standard deviations (HD_95_ = 42.84 ± 50.62 mm; HD_98_ = 61.48 ± 79.77 mm), indicating that while the majority of contours demonstrated reasonable boundary agreement, a subset of physicians produced contours with substantial local deviations.

For the post-MRM case (**Table 3**), all target volumes demonstrated suboptimal inter-observer agreement. Among them, *CTVp_thoracic wall* achieved the highest DSC (0.63 ± 0.08), followed by *CTVn_L4* (0.57 ± 0.15) and *CTVn_IMN* (0.42 ± 0.14). In contrast, *CTVn_L3* exhibited the poorest consistency, with its DSC dropping to 0.38 ± 0.26. The large standard deviations observed across most HD metrics further reflected the substantial inter-observer variability for these anatomically challenging structures.

### 3.3 Qualitative analysis

**Figure 1:**
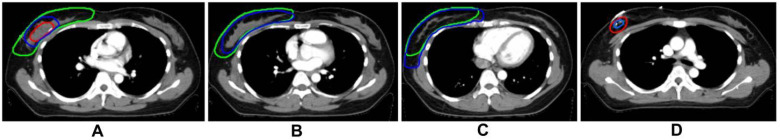
Representative examples of common target volume delineation errors following breast-conserving surgery. **(A)** Consensus reference standard contours established by senior experts, color-coded as follows: Red = tumor bed (*CTV_tb*); Blue = tumor bed boost (*CTV_boost*); Green = whole breast clinical target volume (*CTVp_breast*). **(B)** Inadequate anterior margin of the whole breast *CTVp_breast* (blue lines from participating physicians), failing to reach 5 mm beneath the skin surface. **(C)** Medial and lateral over-contouring of the whole breast *CTVp_breast* (blue lines), extending beyond the edge of the sternal bone medially and the lateral breast fold laterally. **(D)** Under-contouring of the focal *CTV_tb* (blue lines) compared to the expert red contour.

**Figure 2:**
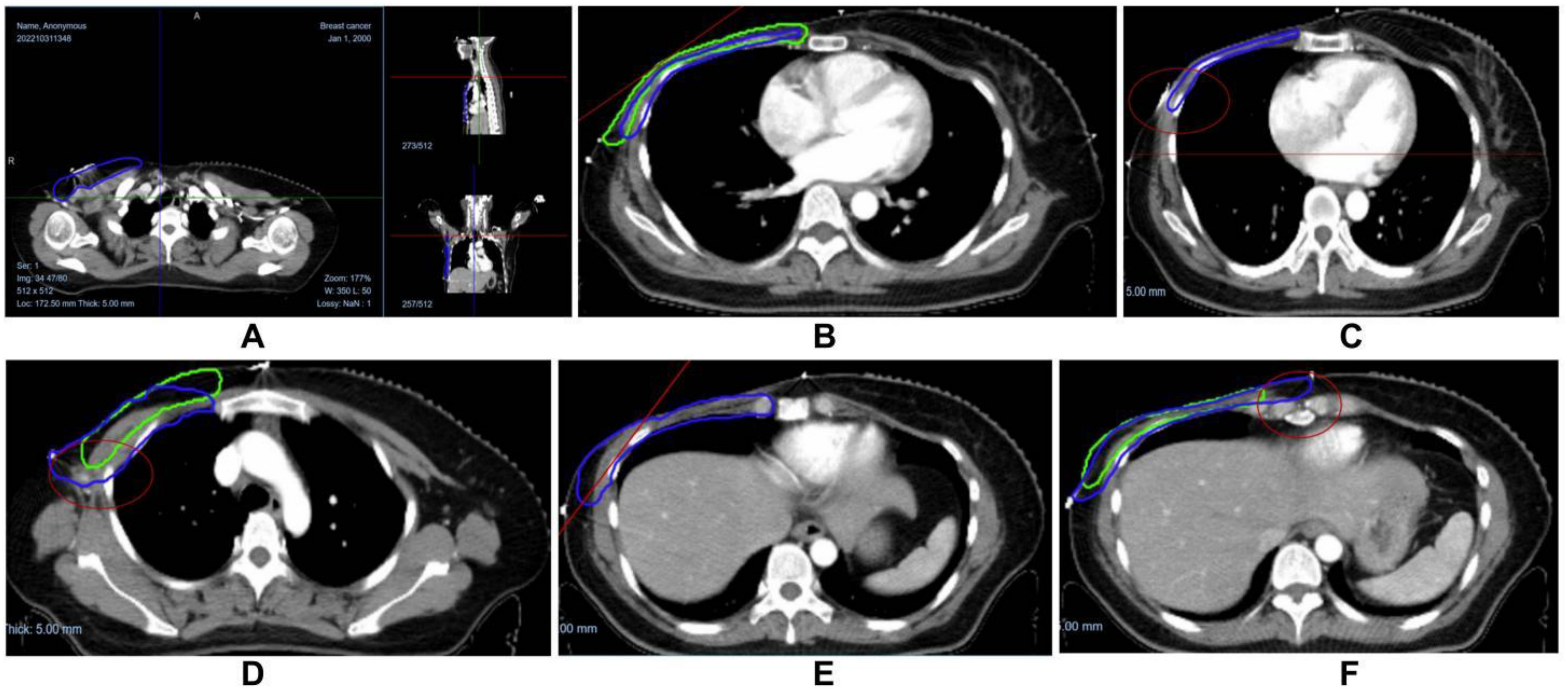
Representative examples of common *CTVp_thoracic wall* delineation errors following modified radical mastectomy. Note: The expert consensus reference standard for the chest wall (*CTVp_thoracic wall*) is displayed as a solid green outline, while individual contours from the participating regional physicians are represented by solid blue outlines. **(A)** Superior border extending beyond the inferior margin of the clavicular head. **(B)** Inadequate anterior margin, failing to completely encompass the skin surface. **(C)** Lateral under-contouring, failing to reach the usually defined anterior to the mid-axillary line boundary. **(D)** Lateral over-contouring, extending beyond the mid-axillary line. **(E)** Posterior over-contouring, extending beyond the major pectoral muscle, costae, or intercostal muscles into the lung parenchyma. **(F)** Medial over-contouring extending beyond the edge of the sternal bone.

**Figure 3:**
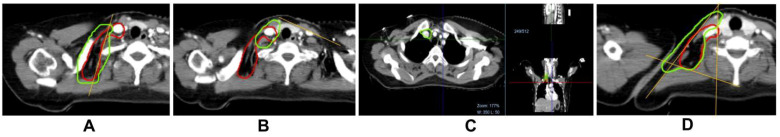
Representative examples of common *CTVn_L4* delineation errors following modified radical mastectomy. Note: The expert consensus reference standard for the supraclavicular nodes (*CTVn_L4*) is denoted by a thick red outline, while the variant boundaries delineated by the participating regional physicians are represented by thin green outlines. **(A)** Lateral and posterior over-contouring (extending beyond the anterior scalene muscles), combined with medial under-contouring (failing to reach the internal jugular vein). **(B)** Inferior over-contouring, extending below the connection to the cranial border of *CTVn_IMN*. **(C)** Anterior over-contouring, extending beyond the sternocleidomastoid muscle or the dorsal edge of the clavicle. **(D)** Both medial and lateral over-contouring.

Qualitative visual review of the representative contouring deviations (**Figures 1**–**3**) corroborates the quantitative metric discrepancies and reveals distinct, systematic patterns of anatomical misinterpretation among the participating physicians.

For the post-BCS targets (**Figure 1**), distinct directional biases were observed between the whole breast and tumor bed volumes. Specifically, for the whole breast target volume (*CTVp_breast*), 75.0% (15/20) of participating physicians demonstrated inadequate anterior margins, failing to maintain the required 5 mm depth from the skin surface. Additionally, over-contouring was frequently observed at the bony and anatomical boundaries: 70.0% (14/20) of physicians extended the medial border beyond the edge of the sternal bone, and 70.0% (14/20) extended the lateral border beyond the lateral breast fold. Conversely, for the tumor bed (CTV_tb), 88.2% (15/17) of the physicians systematically under-contoured the volume, maintaining boundaries tighter than the reference standard.

For the post-MRM targets, qualitative visual analysis identified distinct, border-specific delineation errors across the chest wall and regional nodal stations.

For the thoracic wall target volume (*CTVp_thoracic wall*, **Figure 2**), boundary deviations were predominantly concentrated at the peripheral horizontal borders. Specifically, 85.0% (17/20) of the participating physicians demonstrated medial over-contouring extending beyond the edge of the sternal bone, and 80.0% (16/20) demonstrated lateral over-contouring extending beyond the mid-axillary line. Conversely, vertical, anterior, and posterior deviations occurred less frequently: 40.0% (8/20) of physicians produced inadequate anterior margins that failed to completely encompass the skin surface, 35.0% (7/20) extended the superior border beyond the inferior margin of the clavicular head, and 30.0% (6/20) committed posterior over-contouring by extending beyond the major pectoral muscle, costae, or intercostal muscles into the lung parenchyma. Additionally, instances of lateral under-contouring were observed, where contours failed to reach the defined anterior to the mid-axillary line boundary.

For the supraclavicular nodal station (*CTVn_L4*, **Figure 3**), visual inspection revealed distinct patterns of boundary variation relative to the consensus reference standard. Delineation errors involved lateral and posterior over-contouring extending beyond the anterior scalene muscles, frequently combined with medial under-contouring that failed to reach the internal jugular vein. Furthermore, physicians demonstrated anterior over-contouring extending beyond the sternocleidomastoid muscle or the dorsal edge of the clavicle, inferior over-contouring extending below the junction to the cranial border of the internal mammary nodal volume, as well as simultaneous over-contouring at both the medial and lateral boundaries.

## 4 Discussion

The widespread adoption of intensity-modulated radiotherapy (IMRT) for breast cancer has substantially improved target coverage and dose conformity compared with conventional techniques (18). However, this technical advancement places heightened demands on the geometric accuracy of CTV and OAR delineation, requiring thorough anatomical knowledge and considerable contouring expertise from radiation oncologists (19). While DL-based auto-segmentation has shown promise for OARs with well-defined tissue boundaries, its performance for breast cancer CTVs remains limited due to individual anatomical variations — including differences in surgical approach, breast morphology, and soft tissue interfaces (20, 21). Manual CTV delineation, therefore, remains indispensable, and understanding its inter-observer variability is essential for quality improvement (22). The present study quantified such variability among regional radiation oncologists and revealed significant inconsistencies, particularly for post-MRM nodal target volumes.

### 4.1 Analysis of post-BCS contouring errors

Quantitative evaluation of the post-BCS case revealed a volume-dependent variance between volumetric overlap (DSC) and geometric boundary precision (HD). The mean DSC for the whole breast CTV (*CTVp_breast*) was 0.80, exceeding the threshold of 0.70 generally considered to indicate adequate spatial overlap (23). Yet, it exhibited wider geometric boundary deviations and variability (HD_98_ = 61.48 ± 79.77 mm). This surface distance discrepancy was driven by the documented frequencies of inadequate anterior margins and peripheral over-contouring at the medial and lateral boundaries. That the overall DSC remained high despite these localized boundary variations highlights the inherent volume-dependency of these metrics. For large structures like the whole breast, peripheral errors can be diluted by the volume of the overlapping central region. In contrast, the tumor bed (*CTV_tb*), a smaller structure, yielded a lower DSC (0.56 ± 0.19) but demonstrated a closer boundary fit (HD_95_ = 11.51 ± 6.69 mm). This phenomenon corresponds to the systematic under-contouring of the tumor bed observed across the cohort. Because the baseline volume of the *CTV_tb* is small, millimeter-level spatial shifts reduce the DSC, even when the surface distance remains small.

### 4.2 Analysis of post-MRM contouring errors

Delineation compliance and geometric agreement across all evaluated target volumes were characterized by volume-dependent dilution effects in primary targets, high consistency in anatomically preserved nodal regions, and pronounced clinical decision-making divergence in high-risk or obscured nodal stations.

For primary targets, a clear variance emerged between post-BCS and post-MRM volumes. In *CTVp_breast*, the larger intact tissue volume diluted peripheral errors, resulting in a high DSC (0.80). Conversely, surgical removal of the breast tissue reduces the baseline volume of *CTVp_thoracic wall* compared to an intact breast. This reduction of the volumetric dilution effect lowered its DSC (0.63 ± 0.08), while its geometric boundary deviations maintained considerable variability.

Among post-MRM regional nodal structures, the supraclavicular station (*CTVn_L4*) achieved a high completion rate, second only to the thoracic wall, with a stable agreement metric (0.57 ± 0.15). This compliance stems from two factors: first, *CTVn_L4* remains outside the standard scope of axillary dissection, leaving its native tissue planes and landmarks—such as the sternocleidomastoid muscle, anterior scalene muscle, and internal jugular vein—intact and radiographically distinguishable; second, postoperative *CTVn_L4* irradiation carries a consensus-driven recommendation across major guidelines for node-positive disease, minimizing therapeutic ambiguity (13, 14).

In contrast, completion variances in other nodal stations indicate substantial clinical and technical uncertainty. The low completion rate for internal mammary nodes (*CTVn_IMN*: 30.0%) reflects a therapeutic dilemma regarding survival benefit versus late cardiopulmonary toxicities (24, 25), compounded by a lack of radiographic contrast separating small internal mammary vessels from fat pads on non-contrast CT. Crucially, the geometric metrics for such low-completion target volumes were calculated exclusively from the subset of participants who performed the delineation (N = 6). This presents an inherent selection bias that may overstate the cohort-wide agreement, as non-completing participants are mathematically excluded from these scores. Similarly, the reduced completion of axillary Level III (*CTVn_L3*) reflects a pattern of omission driven by anatomical ambiguity, as surgical flattening and tissue fibrosis obscure traditional landmarks, leading clinicians to exclude these volumes to protect adjacent critical structures (13).

### 4.3 Clinical implications

The findings of this study offer an empirical roadmap for regional practitioners. First, there is a recognized need to conduct systematic learning regarding clinical target volume delineation. During this process, attention should be paid to the boundary definitions of large-volume targets, such as *CTVp_breast* and *CTVp_thoracic wall*, to control peripheral variations at the medial, lateral, and skin extensions. Conversely, for small focal structures like the tumor bed *CTV_tb*, systematic training should focus on addressing under-contouring tendencies associated with small baseline volumes and subsequent variations in peripheral coverage. Furthermore, continuous education should place particular emphasis on target volumes that are frequently omitted in routine practice, such as the internal mammary nodes *CTVn_IMN* and axillary Level III *CTVn_L3*, to reduce the clinical uncertainty arising from post-surgical anatomical alterations and toxicity concerns. Finally, implementing targeted, case-based delineation workshops is warranted to support the translation of these guidelines into uniform clinical decisions.

### 4.4 Limitations

Several limitations of this study should be acknowledged. First, the evaluation was based on a single representative patient case pair, which may limit the direct generalizability of the findings to broader anatomical variations. Second, the consensus reference standard was established by senior radiation oncologists from a single institution, which may partially reflect localized contouring preferences. Third, while the number of participating physicians was sufficient to identify general trends, the sample size limits the statistical precision of the reported geometric metrics. Fourth, granular demographic profiles—such as specific years of practice or subspecialty experience—were not collected, preventing experience-based subgroup stratification. Finally, this baseline study lacks direct dosimetric validation due to the logistical burden of planning 40 distinct multi-volume observer contour sets. Future multi-center initiatives incorporating larger physician cohorts, diverse patient anatomies, multi-institutional reference standards, and automated dosimetric evaluations are warranted to extend these findings.

## 5 Conclusions

Inter-observer consistency in CTV delineation was satisfactory for the whole breast following BCS but was suboptimal for all post-MRM target volumes — particularly the regional nodal stations, where both delineation consistency and completion rates were poor, characterizing the practice patterns of radiation oncologists in non-specialized, community-based settings. These findings highlight the critical need for targeted training programs emphasizing cross-sectional lymphatic anatomy, structured contouring workshops, and the implementation of institutional peer-review quality assurance processes. Such initiatives are expected to reduce inter-observer variability, enhance dosimetric agreement, and ultimately contribute to improved therapeutic outcomes in postoperative breast cancer radiotherapy.

## Data Availability

The original contributions presented in the study are included in the article/supplementary material. Further inquiries can be directed to the corresponding author.
